# Specific identification of *Mycobacterium bovis* by Loop-Mediated Isothermal Amplification (LAMP) targeting the Region of Difference 12 (RD12) of the *M. tuberculosis* complex

**DOI:** 10.1016/j.mex.2023.102223

**Published:** 2023-05-18

**Authors:** Alejandro Sierra, Danna Camelo, Camila Lota, Nelson Enrique Arenas, Carlos Y. Soto

**Affiliations:** a Chemistry Department, Faculty of Sciences, Universidad Nacional de Colombia, Ciudad Universitaria, Carrera 30 N° 45-03, 111321, Bogotá, Colombia; b Biology Department, Faculty of Sciences, Universidad Antonio Nariño. Carrera 1 Este #47a-15, Bogotá, Colombia; c Faculty of Agricultural Sciences, Universidad de Cundinamarca, Diagonal 18 No. 20-29, Fusagasugá, Colombia

**Keywords:** Loop-Mediated Isothermal Amplification (LAMP) of M. bovis targeting the RD12 genomic region, Mycobacterium bovis, RD12 genomic region, M. tuberculosis complex, LAMP-PCR, Phenol red colorimetric reaction, point-of-care method

## Abstract

Bovine tuberculosis is a prevalent zoonotic disease that causes high risks for production animals, dairy producers and consumers, together with significant economic losses. Thus, methods for easy, fast and specific detection of *Mycobacterium bovis* in small and medium-sized livestock under field conditions are very required. In this work, a Loop-Mediated Isothermal Amplification LAMP-PCR targeting the Region of Difference 12 (RD12) of *M. bovis* genome was designed for the purpose of identification. A set of six primers designed for the isothermal amplification of five different genomic fragments led to the specific identification of *M. bovis* from other mycobacterial species. A basic colorimetric reaction was clearly observed at first sight under natural light, indicating positive identification of *M. bovis* in a maximum of 30 min of isothermal amplification at 65 °C. The limit of detection was near 50 fg of *M. bovis* genomic DNA, corresponding approximately to 10 copies of the genome.

• The proposed LAMP-PCR amplification of M. bovis genomic DNA might be performed by untrained laboratory personnel.
• Specific identification of M. bovis LAMP is possible in 30 min at 65.. C using a simple water bath.
• The basic colorimetric reaction for M. bovis identification could be observed with the naked eye under natural light.

## Graphical abstract

**Figure:**
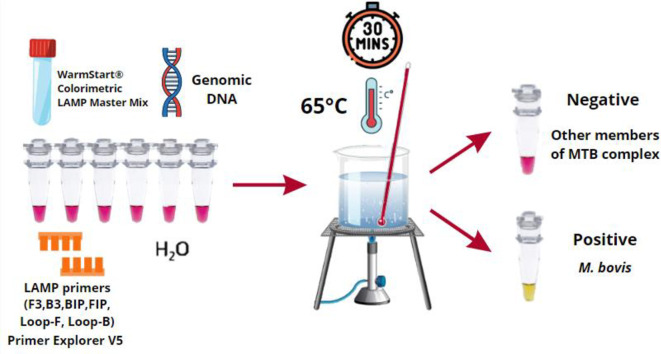

Specifications table

**Table:** 

Subject area	*Biochemistry, Genetics and Molecular Biology*
More specific subject area	*Molecular mycobacteriology*
Name of your Methdod	*Loop-Mediated Isothermal Amplification (LAMP) of M. bovis targeting the RD12 genomic region*
Name of your method	*N/A*
Resource availability	*N/A*

## Methods details

### Introduction

Bovine tuberculosis (BTB) is a zoonotic disease caused by the acid-fast bacilli *Mycobacterium bovis* which threatens livestock safety [1]. According to the World Organization for Animal Health (WOAH), BTB is a reportable disease as stated in the Terrestrial Animal Health Code [2]. Although cattle are considered the primary host of *M. bovis*, buffaloes are also a vital reservoir together with goats, sheep, pigs, horses, camelids, and pets [3]. BTB is spread through contact between infected animals and a susceptible host. Transmission generally occurs by direct contact with ill animals since bacilli are excreted in exhaled droplets into saliva, milk, urine, feces, semen, and vaginal and uterine secretions [4]. BTB spreads through the body in two stages: the primary complex and post-primary dissemination, where the primary complex consists of the initial lesion at the local lymphatic system. In some animals, a reactivation of the latent infection may occur causing progressive tissue damage and a granuloma that increases over time and forms a chronic lesion or subsequent primary diffusion [5].

An animal positive for BTB must be euthanized to prevent low-quality products from reaching the final consumer, and to avoid economic losses as well as the need to immobilize the rest of the herd [2,6]. Hence, early detection of BTB is pivotal to ensure effective control of any outbreak, and minimize the economic impact on farms [7]. The gold standard methods for BTB diagnosis are based on cellular immunity through the intradermal tuberculin skin test (TST) and/or the interferon-gamma release assay (IGRA) [8].

The WOAH and the European Commission established the tuberculin test as the official proof for BTB diagnosis [9]. However, tuberculin shows constraints regarding specificity and sensitivity (from 68 to 95%) affecting the effectiveness of the surveillance system [10,11]. Another diagnostic method developed is the IFN-γ release auxiliary assay that, through ELISA, detects and quantifies the release of the IFN-γ cytokine. Based on this assay, a cell-mediated immune response may be detected after two weeks, in the late stages of active infection [12,13]. A further method is the enzyme-linked immunospot (ELISpot) for BTB diagnosis using bio-labeled IFN-γ 3E9 and 6F8 monoclonal antibodies based on their capture and detection [14].

Despite enhanced sensitivity of around 81.8% and specificity of up to 94%, this method is still limited in detecting infection at different stages, as well as in postmortem tests [15]. On the other hand, molecular-based tools such as mycobacterial interspersed repeat units-variable number tandem repeats (MIRU-VNTR), spoligotyping, and restriction fragment length polymorphism (RFLP) are currently proposed for mycobacterial DNA detection. However, their implementation might be expensive and require sophisticated equipment [16]. This issue entails the challenge of implementing an accurate diagnostic method allowing timely therapeutic intervention, a reduction of deterioration in animal health, and control of infectious diseases under field conditions. In response to this challenge, some isothermal amplification methods to identify *M. bovis* have been successfully proposed to overcome the limitations of PCR-based methods in different settings [[17], [18], [19]]. Thus, Loop-Mediated Isothermal Amplification PCR (LAMP-PCR) turns to be an alternative of interest for the specific identification of *M. bovis.* Through LAMP-PCR, it is possible to obtain nucleic acid amplification at a constant temperature (60–65 °C) using normally 4–6 primers that specifically amplify 6–8 different genomic regions. As the DNA polymerase normally used in LAMP has a high capacity of displacement, the products of the amplification can be detected with the naked eye using DNA binding dyes [20]. Despite the fact that LAMP-PCR requires the designing of a complex set of primers, this technique is highly sensitive and specific, and may be comparable, in terms of efficacy, to other methods of nucleic acid amplification such as: Nucleic Acid Sequence-Based Amplification (NASBA); Recombinant Polymerase Amplification (RPA); or Signal-Mediated Amplification of RNA Technology (SMART) [20]. In addition, unlike NASBA and SMART, LAMP does not need RNA to initiate reaction, nor several enzymes to perform reactions as are required in RPA, which increases costs, precautions towards the RNA, and time [19,20]. These characteristics, together with its capacity of amplification, and the no need of specialized personnel, make LAMP-PCR an ideal technique to implement in the eventual development of a point-of-care diagnosis test for the identification of *M. bovis*.

Also, *M. tuberculosis* complex (MTC) is composed of bacterial species that share identical 16S rRNA sequences [21] and that have more than 99.9% of identity in their genome [22]. However, variable regions resulting from insertion-deletion events have been found in different species of the complex. Most of these polymorphisms did not occur independently, but were rather the result of ancient and irreversible genetic events [23]. Genomic analyses have led to the identification of at least 20 deletions known as Region of Difference (RD) that accumulated within the MTC [23,24]. Thus, it is possible to assert that RDs are suitable genomic regions for the specific identification and differentiation of MTB complex species. In particular, the RD12 is present in the *M. bovis* genomes but absent in the other members of the MTC [24], therefore it is a potential target for *M. bovis* identification by using nucleic acid amplification techniques.

Considering that the long-term objective of this research is the development of a point-of-care method, the use of an isothermal amplification technique allows simplifying the protocols and applying molecular diagnosis outside specialized laboratories. In brief, the purpose of this paper was to develop a specific test to identify *M. bovis* isolates by amplification, based on the Region of Difference 12 (RD12), and using a Loop-Mediated Isothermal Amplification PCR (LAMP-PCR).

## Materials and methods

### Bacterial strains and growth conditions

The mycobacterial strains used in this work are listed in Table 1. *M. bovis*. ATCC 27,290 reference strain was provided by the Colombian Agricultural Institute (ICA); the rest of mycobacterial strains were provided by the National Institute of Health (INS) of Colombia. Mycobacteria were cultured in Middlebrook 7H9 liquid broth supplemented with ADC (0.5% Bovine albumin Fraction V, 0.2% dextrose, and 0.004% catalase) and sodium pyruvate 40 mM at 37 °C with agitation (80 rpm).

**Table 1:** Mycobacterial strains used in this study.

	Bacterial strains	ATCC Reference
	*Mycobacterium bovis*	27,290
	*Mycobacterium bovis* (clinical isolate)	N/A
	*Mycobacterium tuberculosis* H37Rv	27,294
	*Mycobacterium bovis* BCG	35,734
	*Mycobacterium avium*	19,698
	*Mycobacterium fortuitum*	2008 32,023
	*Mycobacterium absessus*	CCUG 48,898

### Mycobacterial DNA isolation

10 mL of stationary phase culture was centrifuged at 8500 rpm for 15 min. Pellet was resuspended in 500 µL of 1X TE solution (10 mM Tris–HCl 10 mM EDTA, pH 8.0) and heated at 85°C for 20 min. Subsequently, lysozyme (10 mg/µL) was added and incubated at 37 °C for 8 h. Then, SDS (10%) and proteinase K (10 mg/µL) was added and samples were incubated during 1 h at 65 °C. Next, NaCl (4 M) and CTAB/NaCl preheated to 65 °C were added following incubation at the same temperature during 45 min additional. After that, one volume of isoamyl alcohol: chloroform (24:1) was added and samples were vortexed and centrifuged at 13,000 rpm for 10 min at room temperature. The aqueous phase was transferred, RNAse (20 mg/µL) was added and samples were incubated at 37 °C for 15 min. The isoamyl alcohol: chloroform extraction and centrifugation were repeated. The aqueous phase was transferred to a new tube and supplemented with 100 uL of 3 M sodium acetate; then, one volume of isopropyl alcohol was added followed by incubation at −20 °C overnight. Then, samples were centrifuged at 13,000 rpm for 15 min at 4 °C; the supernatant was discarded; two volumes of ethanol were added; samples were incubated at −20°C for 1 h; and DNA was precipitated by centrifugation at 13,000 rpm for 15 min. DNA was washed twice with 70% ethanol discarding supernatant. Finally, samples were dried at 37 °C for 30 min and pellets were resuspended in TE buffer (1X). DNA integrity was evaluated by agarose gel electrophoresis (1%) in TAE 1X Buffer (Tris-acetate-EDTA: 40 mM Tris, 20 mM Acetate and 1 mM EDTA, pH 8.3), and quantified in a NanoDrop OneC UV–Vis Spectrophotometer (Thermo Scientific, MA, USA).

### Primer design and LAMP-PCR standardization

A set of 6 primers (outer forward-F3, outer primer reverse-B3, inner primer forward-FIP, inner primer reverse-BIP, loop primer forward-FLP, and loop primer reverse-BLP, Table 2) were designed within the RD12 region of the *M. bovis* genome (http://genolist.pasteur.fr/BoviList/)*,* using the Primer ExplorerV5 software (http://primerexplorer.jp/lampv5e/index.html) Firstly, F3, B3, FIP and BIP primers were designed according to the parameters set by ExplorerV5 in order to optimize primer parameters and to improve reaction specificity. Primers were evaluated by using the Primer-BLAST tool (https://www.ncbi.nlm.nih.gov/tools/primer-blast/) and, *in silico*, by using PCR (http://insilico.ehu.es/PCR/) to assess the closest species and prevent undesired priming [25]. *In silico* assays preliminary showed potential false positives in some amplification reactions with certain species of the MTC. Consequently, the loop primers (FLP and BLP) were also designed using ExplorerV5 with the purpose of increasing the specificity of the amplification reactions [26].

**Table 2:** Primer sequences targeting the RD12 region in *M. bovis* for the LAMP-PCR*.* Concentrations were included in µM.

Primer	Sequence (5′-3′)	Primer Concentration
FIP	GAACCAGGTGTGCGAGGACC-AGACAACAGCAAGGAAACGA	1.6
BIP	TGTTGCGGGAATTACTCGGACA-GGCAAGCATGTGGACCATA	1.6
F3	TTGGCCAAGCTTTACGCC	0.2
B3	GCGTGAATACGCTGAGGC	0.2
LOOP-F	CCGATTCGGCAGTAGGCAA	0.4
LOOP-B	CCAAAACGTCAACATAGCATTTGG	0.4

LAMP-PCR reactions were optimized using the WarmStart® Colorimetric LAMP 2X Master Mix (DNA & RNA) (New England Biolabs, Ma, USA) according to the manufacturer's recommendations, with some minor modifications. Thus, 25 µL final volume of reactions was performed in 200 µL Eppendorf tubes containing (Table 3): target DNA (variable concentrations), LAMP Master Mix (Bst 2.0 WarmStart DNA Polymerase, WarmStart RTx Phenol Red for pH detection of LAMP, and 8.0 mM MgSO_4_, New England Biolabs, Ma, USA); primers (concentrations recommended by manufacturers, Table 2) and ddH_2_O. Each reaction was incubated in a water bath at 65 °C for 30 min. Reactions supplemented with *M. bovis* ATCC 27290 reference strain DNA were positive controls; and reactions supplemented with no DNA were negative controls. Positive reactions were evaluated colorimetrically, estimating the coloration change of the sample from pink to yellow, which indicates DNA amplification, while in negative reactions the coloration remained pink. These reactions were performed in duplicate in three independent experiments.

**Table 3:** Reagents required for a single LAMP-PCR test.

Reagent	Volume (µL)
WarmStart® Colorimetric LAMP 2X Master Mix	12,5
LAMP Primer Mix 10X	2,5
dH2O	9
Target DNA	1
**Total volumen**	25

### Sensitivity and specificity assays

In order to evaluate the LAMP-PCR sensitivity, reactions were performed using serial dilutions of *M. bovis* ATCC 27290 from 250 ng/µL to 5 fg/µL. On the other hand, to assess specificity, reactions were performed at a DNA concentration of 50 pg/µL, which represents the average concentration found in smear-positive sputum [18,27,28], using genomic DNA of the following mycobacterial species: *M. tuberculosis* (*Mtb*) H37Rv; *M. avium; M. fortuitum; M. abscessus; M. bovis* ATCC 27290; *M. bovis* BCG; and *M. bovis* clinical isolates.

### Method validation

The diagnosis of BTB traditionally depends on methods based on cellular immunity that use the tuberculin skin test (TST) and/or the interferon-gamma release assay (IGRA), together with recent antibody-based methods [3]. Also, molecular-based tools have been developed for the specific detection of *Mtb* complex genomic DNA regions, such as interspersed repeat units-variable number tandem repeats (MIRU-VNTR), spoligotyping, and RFLP. Specifically, PCR-based techniques have several advantages in terms of speed, which means rapid diagnosis and efficient control. Despite the fact that PCR-based techniques require specialized equipment and qualified staff to obtain reliable results, those techniques overcome the specificity limitations of traditional methods used for mycobacteria typing such as cultures or biochemical tests [29]. Nevertheless, molecular techniques based on PCR are expensive, and sometimes, in the case of zoonotic infections, are difficult to perform there where livestock production centers are located, especially in developing countries. Alternatively, point-of-care diagnostic techniques have been proposed as an option to overcome these drawbacks as they can be easily implemented in small and medium-sized livestock centers. In this sense, LAMP has been suggested as a rapid and low-cost method for the detection and surveillance of *M. bovis* in livestock and humans in resource-limited endemic areas where only basic laboratories are available [17,30]. Previously, Kapalamula et al. developed a LAMP method for the specific identification of *M. bovis* by DNA amplification of the RD4 genomic region of the *M. bovis* complex [17]. In that work, positive reactions are detected by fluorescence after applying ultraviolet light to the amplification reaction by using fluorometer equipment, which obviously increases the need for specialized laboratory equipment.

**Fig. 1:**
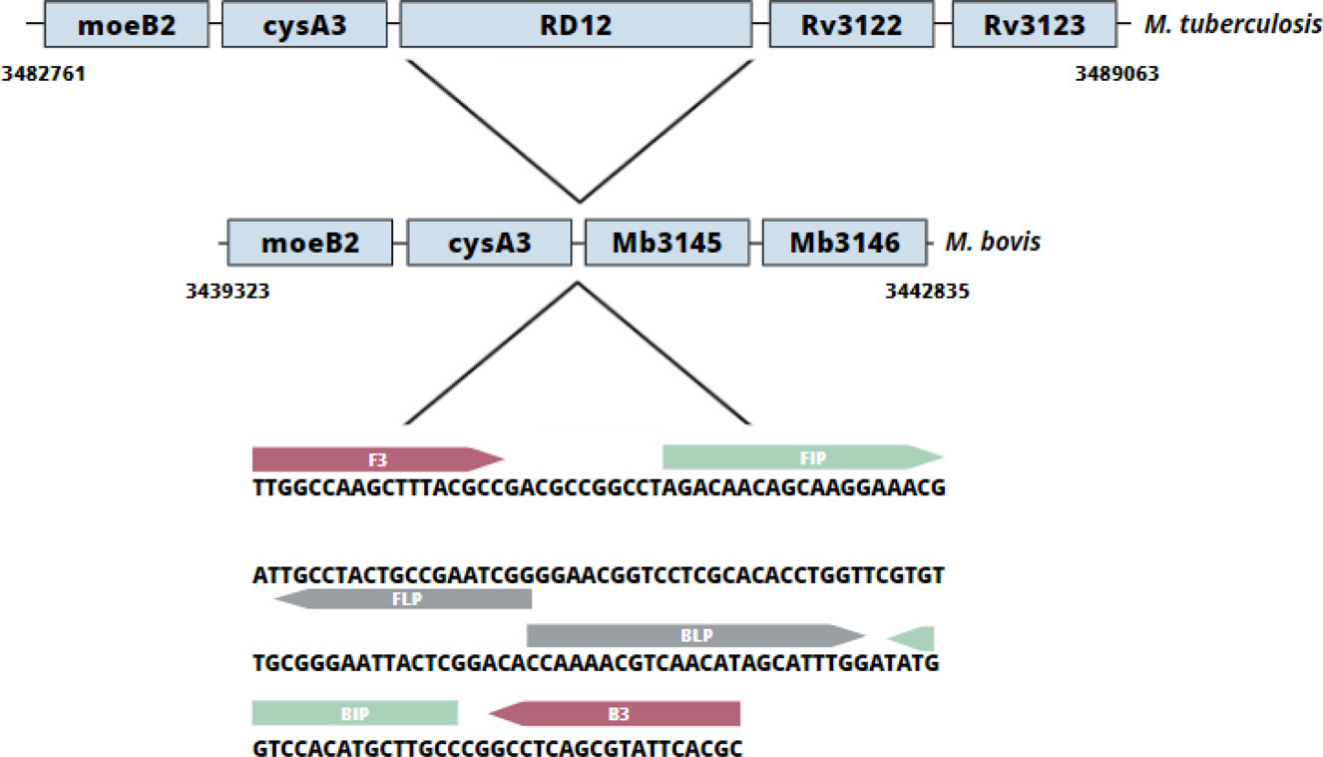
Schematic representation of the Region of Difference 12 (RD12) and design of LAMP-PCR primers.

**Fig. 2:**
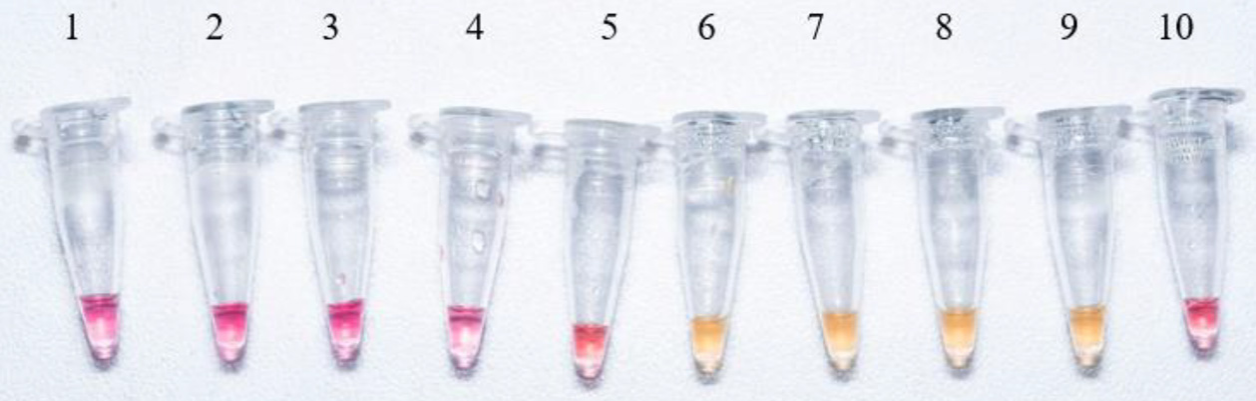
LAMP-PCR reaction specificity test using RD12 primers. From left to right: **1**. *M. tuberculosis* H37Rv; **2**. *M. tuberculosis* H37Rv (isolate); **3**. *M. avium*; **4**. *M. fortuitum*; **5**. *M. abscessus*; **6**. *M. bovis* ATCC 27290; **7**. *M. bovis* BCG; **8**. *M. bovis* (clinical isolate 4609*)*; **9**. *M. bovis* (clinical isolate); and **10**. Negative control (without DNA). Reaction samples were incubated at 65 °C for 30 min. Reactions were performed in duplicate in three independent experiments.

In this study, the RD12 genomic region of the *Mtb* complex is proposed as an alternative target for the specific detection of *M. bovis* by LAMP-PCR. Since *Mtb* also produces BTB, the specific designed set of six primers (Fig. 1) match within specific genomic portions of RD12 present in the *M. bovis* genome, but is absent in the *Mtb* genome. Thus, the proposed primers were useful for the specific differentiation between those *Mtb* complex strains. BTB caused by *M. bovis* is indistinguishable from that which is caused by *Mtb*, and differentiation between them is difficult, either by clinical specimens or culture [17,18]. In this case, all reactions containing *M. bovis* DNA were positive, while all other mycobacterial DNA samples were negative. Moreover, no false positives were detected since closely related species were included in the setting such as those within *Mtb* complex (Fig. 2). Hence, different DNA samples from other mycobacterial species validated the method's accuracy. In addition, positive reactions were stable when they were stored at −20 °C for months.

When the LAMP reaction conditions were optimized, the primer concentration initially used followed the manufacturers’ recommendations (WarmStart® Colorimetric LAMP 2X Master Mix, New England Biolabs, Ma, USA) (Table 1). However, primer concentrations three times upper from those recommended were tested as well showing always the same results.

The reaction temperature was always kept at 65 °C so as to respect the correct functioning of the Bst 2.0 WarmStart DNA Polymerase included in the reaction buffer. Change of reaction coloration from red (negative) to yellow (positive) is given by the acidification of reactions detected by phenol red dye as amplification of DNA fragments by Bst 2.0 DNA Polymerase progresses. On the other hand, when the time of reaction was evaluated, positive results were observed after 22 min of incubation at 65 °C. When the amplification time was more than 30 min, the negative *Mtb* samples turned yellow giving false positive reactions and, when the time reaction exceeded 40 min, negative controls without DNA turned false positive reactions (colored yellow). Therefore, optimized reaction conditions were given when reaction samples incubated at 65 °C for 30 min.

**Fig. 3:**
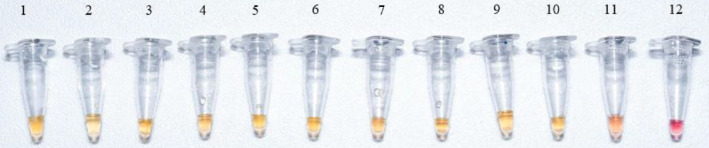
LAMP-PCR reaction sensitivity test using RD12 primers. The amount of DNA in each reaction sample (25 µL) is, from left to right: **1.** 250 ng; **2.** 125 ng; **3.** 50 ng; **4.** 25 ng; **5.** 5 ng; **6.** 500 pg; **7.** 50 pg; **8.** 5 pg; **9.** 500 fg; **10.** 50 fg; **11.** 5 fg; and **12**. Negative control (without DNA). Reactions were performed in duplicate in three independent experiments.

Regarding the sensitivity of the method, the limit of detection of *M. bovis* genomic DNA using the proposed set of primers and reaction conditions was evaluated using serial dilutions of genomic DNA from 250 ng to 5 ng in a reaction sample of 25 µL (Fig. 3). Positive results were clearly observed at first sight under natural light due to the color change of the reaction when the LAMP-PCR was supplemented with up to 50 fg of mycobacterial DNA in each reaction, which is the equivalent to approximately 10 copies of the *M. bovis* genome [17]. Therefore, the isothermal amplification of *M. bovis* DNA using the proposed set of primers is possible under suitable reaction conditions in a wide range of DNA target concentrations, even below the average concentration of a sputum sample.

Although conventional methods like TST and culture are cheap and common, their sensitivity and specificity are limited and then inappropriate for disease surveillance [31]. TST relies on *in vivo* delayed-type hypersensitivity test that reveals infections 3 to 8 weeks after contact with *M. bovis*, so it is not possible to distinguish between active and latent infections. Moreover, TST has low specificity because of cross-reactive responses due to the sharing of many antigens between tuberculous and nontuberculous mycobacteria [32]. On the other hand, even though *M. bovis* culture is considered the gold standard for BTB diagnosis, it is time-consuming and requires specialized equipment and trained staff. Since *M. bovis* is a slow-growth mycobacteria, it requires up to 20 days to obtain colonies, therefore, culture results may take 3 to 4 weeks [33]. In order to overcome the limitations of conventional tests, the LAMP protocol proposed in this work is rapid, sensitive, specific, and allows the amplification of the target at a constant temperature in a single step without the necessity of expensive equipment or sophisticated technical skills. The LAMP trial targets a unique region of the *M. bovis* genome permitting the detection of this specific mycobacteria in animal samples. The test gives results in less than an hour, which means that it is a valuable tool for BTB eradication in surveillance programs. Nevertheless, the LAMP protocol proposed here has some limitations that include the need for specialized reagents and a good quality DNA template. Despite these limitations, the LAMP method has proven to be an effective tool for the diagnosis of BTB in various animal species, and has the potential to improve accuracy and speed in the BTB diagnosis. Hence, the cost-benefit of LAMP technique implementation for BTB diagnosis in animals must be considered since it can improve disease surveillance, facilitate trade, and reduce economic losses in the livestock industry.

BTB incidence leads annually to the slaughter of many animals harboring zoonotic pathogens, and possibly low-quality products are reaching final consumers. Thus, the major problem is still the early detection of the disease in order to ensure the effective control of outbreaks and the food safety [34]. In this work, it is shown that the RD12 region represents a molecular signature for the specific identification of *M. bovis* among the species of the *Mtb* complex as confirmed with other PCR-based methods [35]. The proposed LAMP-PCR method can be used in resource-limited conditions, considering infrastructure and the availability of specialized technical equipment or staff. The reaction conditions could facilitate the assembly of the test tube, and the incubation at a constant temperature, which does not require the use of expensive equipment as a water bath is enough. From the perspective of a complete surveillance strategy [36], the authors of this research previously developed an interactive panel tool to collect testing in BTB outbreaks or hotspots with the purpose of strengthening disease eradication plans in Colombia.

## Conclusions

The RD12 genomic region of the *Mtb* complex is useful to specifically identify *M. bovis* from *Mtb* (which also produces BTB infection in animals), and other mycobacterial species. The set of six primers designed in this work (FIP, BIP, F3, B3, LOOP-F, LOOP-B) was successful for the specific and isothermal amplification of *M. bovis* DNA at 65 °C during 30 min, until a limit of detection of 50 fg genomic DNA of *M. bovis* /reaction. The results of the assay showed specificity and high sensitivity, which constitutes a simple and fast technique, suggesting that this LAMP-PCR reaction could have feasible applicability and possibilities of evaluation in the field when more sophisticated resources are not available. This potential point-of-care test might expand the opportunity to meet food safety standards and, in this way, expanding global markets for livestock industry in developing countries.

## Ethics statements

N/A

## CRediT author statement

Conceptualization, A.S., d-S.C., N-E.A. and C.Y.S..; formal analysis, A.S., d-S.C., N-E.A., C-A.L. and C.Y.S.; funding acquisition, C.Y.S. and N-E.A.; investigation, A.S., d-S.C.; methodology, A.S., d-S.C., N-E.A., C-A.L. and C.Y.S.; data curation, A.S., d-S.C., and C-A.L.; supervision, C.Y.S. and N-E.A.; writing—original draft preparation, A.S., d-S.C., N-E.A., and C.Y.S.; writing—review and editing, N-E.A., and C.Y.S. All authors have read and agreed to the published version of the manuscript.

## Declaration of Competing Interest

The authors declare that they have no known competing financial interests or personal relationships that could have appeared to influence the work reported in this paper.

## Data Availability

The data presented in this study are available on request to the correspondence of the author.
